# Complementary and Alternative Medicine for Victims of Intimate Partner Abuse: A Systematic Review of Use and Efficacy

**DOI:** 10.1155/2014/963967

**Published:** 2014-05-28

**Authors:** Luke Duffy, Jon Adams, David Sibbritt, Deborah Loxton

**Affiliations:** ^1^Research Centre for Gender, Health and Ageing, University of Newcastle, Callaghan, NSW 2308, Australia; ^2^Faculty of Health, University of Technology, Sydney, NSW 2007, Australia

## Abstract

*Objectives*. To examine: (i) the extent to which victims of intimate partner abuse (IPA) use complementary and alternative medicine (CAM) and (ii) the effects of CAM on their mental health. *Methods*. Medline, Scopus, and Web of Science were searched for studies measuring the extent of CAM use amongst victims of IPA and trials assessing the impact of CAM on mental health amongst this population. Risk of bias was assessed using the Cochrane collaboration tool. *Results*. No studies measuring the level of CAM use amongst IPA victims, and only three studies assessing the effect of CAM on the mental health of this population were identified. Two studies looked at yogic breathing, while one assessed the effect of music therapy. All three studies showed some beneficial effects; however, each had a small sample, brief intervention period, and no follow-up measurement and were considered to be at high risk of bias. *Conclusions*. The review found little evidence for the benefits of CAM for IPA victims. Findings suggest positive effects of music therapy and yogic breathing; however, methodological limitations mean that these results should be interpreted with caution. It is important that more research into the use and effects of CAM amongst this population are undertaken.

## 1. Introduction

Intimate partner abuse (IPA) is a widespread and serious public health problem. A World Health Organisation review of 48 population-based studies from across the world found that the proportion of women who experience IPA at some time during their lives ranged from 10% in Paraguay and the Philippines to as high as 67% in rural Papua New Guinea and 69% in Managua, Nicaragua [1]. The proportion of women to have experienced IPA in the past 12 months ranged from 1.3% in the United States and 3% in Australia and Canada to 38% in Korea and 52% in the West Bank and Gaza Strip. IPA is associated with a range of negative health outcomes, including chronic mental and physical health conditions, substance abuse, and physical injury [2, 3].

The mental health impacts of IPA are pervasive and long-lasting. Women who have experienced IPA have been found to have higher rates of mild and severe depressive symptoms, anxiety, posttraumatic stress disorder, suicidal tendencies, and impaired social functioning [2, 4, 5]. Zlotnick and colleagues found that women who experienced IPA were significantly more likely to report higher levels of depression and functional impairment and lower levels of self-esteem and life satisfaction five years later than women without IPA [6].

Despite the substantial mental health burden associated with IPA, findings indicate that consultation with mental health services amongst victims is suboptimal. Only around one in three women who report rape from an intimate partner and around one in four who report physical violence, access mental health care for this abuse [7]. Lipsky and Caetano found that victims of IPA were twice as likely as nonvictims to report unmet need for mental health care [8]. These findings emerge despite the fact that women who have lived with abusive partners have been found to access some services more frequently than other women, including higher rates of general practitioner consultations, outpatient hospital visits, accident and emergency department admissions, and prescriptions [5, 9, 10], and suggest a potentially important role for alternate sources of healing.

In recent years, there has been an increasing research focus on the use of complementary and alternative medicine (CAM) [11, 12]—a range of treatments and practices not traditionally associated with the medical profession or medical curriculum, including mind-body techniques, therapies, and herbal and nutrient products [13]. Findings from a number of reviews suggest that the use of CAM products and services is associated with improvements in mental health. For example, one systematic review found strong evidence for the effectiveness of St. John's wort for the treatment of depression and for kava in the treatment of anxiety, and emerging evidence for the use of omega-3 fatty acids and acupuncture in treating depression and anxiety, respectively [14]. Another review examined the use of yoga in the treatment of mood and anxiety disorders. While there were limited studies, and some methodological shortcomings, findings suggest that yoga may be comparable to antidepressant medication in the treatment of depressive disorders and the combination of the two treatments superior to medication alone [15]. The authors also reported that yoga may be more effective than medication for a subgroup of patients with anxiety disorders.

While these findings point to the potential benefits of CAM use for victims of IPA, no systematic review has examined the use, or effectiveness, of CAM amongst this population. The current review had two aims. Firstly, it aimed to determine the extent to which IPA victims make use of CAM products and services. Secondly, it examined whether CAM use leads to improvements in the mental health of victims. In order to do this, we reviewed studies examining the extent of IPA use, as well as randomised controlled trials assessing the impact of CAM on people who have experienced IPA.

## 2. Methods

### 2.1. Data Sources and Searches

Studies were identified by searching electronic databases and scanning reference lists of articles. The database search was applied to Medline, Scopus, and Web of Science, with the last search run on 10 March 2014. Two sets of search terms were used. The first set referred to IPA, while the second referred to CAM. These were combined using the Boolean operator AND. The complete search strategy for Scopus was as follows: (TITLE-ABS-KEY (“spouse abuse” OR “spousal abuse” OR “battered” OR “partner abuse” OR “domestic violence” OR “domestic abuse” OR “partner violence”) AND TITLE-ABS-KEY (“complementary medicine” OR “alternative medicine” OR acupuncture OR massage OR relaxation OR homeopathy OR herbal OR chiropract$ OR mindfulness OR mind-body OR meditation OR yoga OR naturopath$ OR aromatherapy OR osteopath$ OR “music therapy”)).

### 2.2. Selection Criteria

All original studies measuring the extent to which victims of IPA use CAM were eligible for inclusion. All randomised controlled trials (RCTs) or quasi-RCTs studying the effects of CAM on the mental health of people who have experienced IPA were also eligible. Editorials, reviews, and commentaries were excluded. Trials using CAM as the sole treatment or as an adjunct to other treatments were included. No language, publication date, or publication status restrictions were imposed.

### 2.3. Data Extraction and Risk of Bias

One author (LD) extracted data based on predefined selection criteria. Risk of bias was assessed using the Cochrane collaboration tool [19], with assessments made relating to randomisation and concealment of allocation; blinding of participants; personnel and outcome assessment; extent of loss to follow-up; and selective reporting.

## 3. Results

### 3.1. Description of Studies

**Figure 1:**
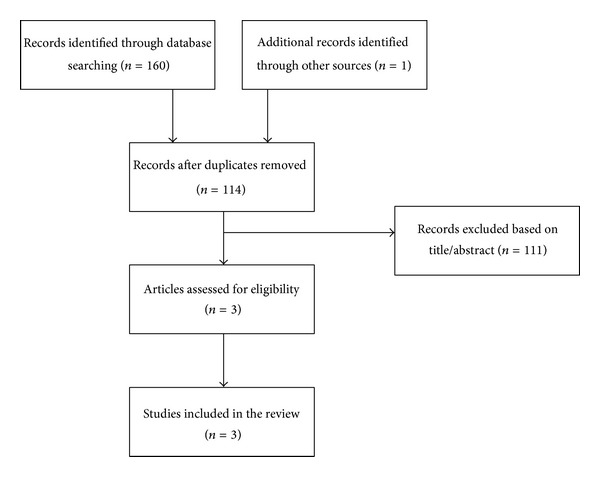
Flowchart of the results of the literature search.

The literature search provided a total of 160 citations, 47 of which were duplicates. Of these, two studies met the selection criteria concerning CAM effectiveness, while another effectiveness study was identified by checking the reference lists of these papers (Figure 1). No studies measuring the level of CAM use amongst IPA victims were identified.

### 3.2. Study Characteristics

*Methods.* One study examined the effect of music therapy on the anxiety levels and sleep patterns of abused women in domestic violence shelters [18]. The music therapy intervention consisted of participant-selected music combined with progressive muscle relaxation for 20 minutes on two consecutive days.

The other two studies investigated the effects of yogic breathing and “giving testimony,” either in combination or individually, on battered women's feelings of self-efficacy [16], and depression [17]. Testimony involved participants describing their experiences of abuse to a trained research assistant. The research assistants were trained to actively listen, asking questions only to encourage participants to elaborate on their experiences.

The yogic breathing intervention involved participants being taught a variety of pranayama techniques by a master's-level student, including regulating the length and depth of inhalation and exhalation, directing the movement of the breath, and using breath with sound. Basic yoga poses were also included in order to enhance the effects of these techniques.

*Participants.* The music therapy study had 28 participants from two centres for battered women. Both yogic breathing studies had 40 participants, half of whom were African-American and the other half were European-American, who were recruited from advertisements.

*Interventions.* The music therapy study was conducted at battered women's shelters and consisted of participant-selected music combined with progressive muscle relaxation for 20 minutes on two consecutive days. The yogic breathing studies also took place over two consecutive days, each involving 45 minutes of learning yogic breathing techniques and 45 minutes of giving testimony (combined condition), or 45 minutes of giving testimony/yogic breathing (individual conditions).

*Control Conditions.* In the yogic breathing studies [16, 17], there was a no-treatment control group, as well as testimony only, and yogic breathing only groups. In the music therapy study, women in the control groups were asked to lie quietly in a darkened room for 20 minutes [18].

*Outcomes*. There were a range of different outcomes assessed across the three studies. The outcomes in the music therapy study were state anxiety, measured by the State Trait Anxiety Inventory (STAI) before and after each music stimulus, and sleep quality measured by the Pittsburgh Sleep Quality Index (PSQI), on the first and last sessions [18]. The outcome in one yogic breathing study was self-efficacy scores, as measured by the Franzblau Self-Efficacy Scale (FSES) 20, taken the day before the intervention began, and the day after it finished [16]. In the other yogic breathing study, the outcome was depression scores as measured by the Beck Depression Inventory II (BDI-II), also taken the day before the intervention began, and the day after it finished [17].

Key data from these studies are summarised in Table 1.

### 3.3. Risk of Bias

Risk of bias evaluations are summarised in Table 2. Information on the method of randomisation and allocation concealment was not provided in either of the yogic breathing studies. In the Franzblau et al. [17] study, pretest scores were much lower in the yogic breathing group and highest in the testimony and combined groups. In the Franzblau et al. [16] study, there were also large differences in pretest scores. This was particularly apparent for the combined testimony/yogic breathing condition, which had the lowest score for three of the five factors, and the second-lowest score on the remaining two factors. In the music therapy study, participants were matched according to their scores on the Pittsburgh Sleep Quality Index, which differentiated between good and bad sleepers, and were assigned to either the experimental or control conditions on an alternating basis as they joined the study. The authors stated that this method was defined a priori in order to avoid researcher bias.

Given the nature of the interventions, blinding of participants and personnel was not possible in any of the three studies. These problems are likely to have been exacerbated in the music therapy study, where the authors reported that participants shared information about treatment. Furthermore, some participants in the control condition of this study reported that lying quietly for 20 minutes was stressful [18]. The use of subjective measurements of participant well-being means that there is a high risk of bias due to lack of blinding of outcome assessment. No participants withdrew from either of the yogic breathing studies, while information about attrition was not provided in the music therapy study. Due to the inadequate blinding of participants, personnel and outcome assessment, all three studies were judged to be at high risk of bias.

### 3.4. Syntheses of Results

Each of the three studies showed positive effects of CAM interventions. In the first yogic breathing study, women in the yogic breathing only condition had significantly improved scores on one of the five self-efficacy factors (unafraid/afraid), while those in the control condition improved on none of these factors [16]. Women in the combined testimony/breathing condition had significantly improved scores on three of the factors (secure/insecure, afraid/unafraid, and confident/not confident), while women in the testimony condition improved on just one factor (afraid/unafraid). In the second yogic breathing study, women in the yogic breathing only condition had a significantly greater reduction in depressive symptoms than women in the control group [17]. In the music therapy study, women in the experimental group had greater reductions in anxiety levels than those in the control condition, and there was a significant interaction by condition on anxiety levels on both days [18]. Those in the experimental group also had significant improvements in sleep quality, while those in the control condition did not.

## 4. Discussion

Evidence concerning the use and effectiveness of CAM use for those who have experienced IPA is limited. Regarding the first aim, the literature search found no studies measuring the extent to which IPA victims use CAM products and services. For the second aim, three studies were identified that examined the effect of CAM on the mental health of this population. While each of the three trials provided preliminary support for the use of CAM (specifically music therapy and yogic breathing) in improving the health of IPA victims, they are limited by small sample sizes and other methodological limitations. There is currently no evidence for the long-term effectiveness of CAM for victims of IPA, while the brief intervention periods (two days in each of the included studies) mean there is also no evidence concerning the prolonged use of CAM.

The lack of research examining the use and effectiveness of CAM amongst victims of IPA is concerning. Given the lack of consultation with mental health services, it is important to know whether victims of IPA use, and benefit from, CAM. It is possible that the stigma of IPA may lead some victims to use CAM as an alternative to mental health care. A study conducted by Prospero and Vohra-Gupta involving college students who had experienced IPA found that embarrassment and social stigma were among the most prominent barriers to seeking help from a mental health professional [20]. Stigma has also been identified as a contributing factor to the higher rates of CAM use found amongst people with depressive symptoms, leading sufferers to use self-chosen treatments that can be accessed outside of conventional health care [21].

It is important that future studies examine the extent to which victims of IPA make use of CAM services, in order to establish the prevalence and characteristics of CAM users. It is also important that more work is done to examine the efficacy of CAM. This work should include larger, more diverse samples, involving participants from a range of racial/ethnic backgrounds and of varying ages and demographic groups, as well as male, and gay and lesbian victims of IPA in order to show evidence for the generalisability of treatment. These studies would also benefit from considerably longer intervention periods, of at least several weeks' duration. Greater delays between completion of the intervention and outcome assessment, as well as follow-up measurements of participant functioning, would also enable stronger conclusions to be drawn about the long-term benefits of CAM. Finally, it is important that future studies use blinded raters and objective, clinician-administered measures in order to minimise the risk of bias in outcome assessment.

### 4.1. Limitations

No studies measuring frequency of CAM use and only three relevant effectiveness studies were identified. All had small sample sizes, brief interventions, and no follow-up measurements.

The two yogic breathing studies consisted of only African-American and European-American women, with five members of each group in each of the four conditions [16, 17].

The limitations of such brief interventions and limited followup were highlighted in the music therapy study. While those in the experimental condition had significant reductions in anxiety symptoms on both days, these effects appear to be short-lived. Despite the large reduction in anxiety symptoms on day one, participants in the experimental group actually had slightly higher STAI scores in the day two of pretest than did those in the control condition.

### 4.2. Conclusion

This systematic review identified no studies measuring the extent to which IPA victims use CAM and only three studies that assessed the effect of CAM on health amongst this population. Findings from the studies suggest that CAM, specifically music therapy and yogic breathing, may be beneficial to people who have experienced IPA; however, methodological limitations mean that these results should be interpreted with caution. It is important that future research measures the uptake of CAM amongst this population, and that more rigorous and methodologically-sound investigations of the effects of CAM are conducted. This work should include larger sample sizes, longer interventions, and extended follow-up periods. Given the enormously damaging health, social and economic consequences of IPA, and an apparent reluctance amongst victims to access mental health care, it is vital that a comprehensive evidence-base of CAM research is established.

## Figures and Tables

**Table 1:** Characteristics of the included studies.

Citation	Intervention	Control group	Participants	Outcomes	Results
Franzblau et al., (2006)* [16]	45 minutes of yogic breathing and/or 45 minutes of giving testimony on two consecutive days	Waiting controls	20 African-American and 20 European-American women, who had been abused by a man with whom they had been intimate with in the past 2 years	Franzblau self-efficacy scale (FSES) 20: (1) anxious/relieved (2) in control/out of control (3) secure/insecure (4) unafraid/afraid (5) confident/not confident	Yogic breathing group had significantly improved unafraid/afraid scores, while control condition did not. Combined condition improved on three factors, while testimony only condition improved on one factor. Neither group improved on any of the other outcomes.

Franzblau et al., (2008)* [17]	45 minutes of yogic breathing and/or 45 minutes of giving testimony on two consecutive days	Waiting controls	20 African-American and 20 European-American women, who had been abused by a man with whom they had been intimate within the past 2 years	Beck depression inventory II (BDI-II)	Yogic breathing group had a significantly greater reduction in depressive symptoms than control group.

Hernández-Ruiz (2005) [18]	Music therapy (participant-selected music combined with progressive muscle relaxation) for 20 minutes on two consecutive days	Lying quietly for 20 minutes on two consecutive days	28 participants from two centres for battered women	(1) State Trait Anxiety Inventory (STAI) (2) Pittsburgh Sleep Quality Index (PSQI)	(1) Significant interaction by condition on anxiety levels on both days. (2) Experimental group had significant improvements in sleep quality, while control condition did not.

*Separate studies using the same methods.

**Table 2:** Risk of bias assessment of the included studies*.

Citation	Random sequence generation	Allocation concealment	Blinding of participants and personnel	Blinding of outcome assessment	Incomplete outcome data	Reporting bias
Franzblau et al., (2006) [16]	Unclear	Unclear	High risk	High risk	Low risk	Low risk
Franzblau et al., (2008) [17]	Unclear	Unclear	High risk	High risk	Low risk	Low risk
Hernández-Ruiz (2007) [18]	Low risk	Low risk	High risk	High risk	Unclear	Low risk

*Based on the Cochrane Collaboration's tool for assessing risk of bias [19].
